# Midazolam plasma concentration after anesthesia premedication in clinical routine - an observational study: Midazolam plasma concentration after anesthesia premedication

**DOI:** 10.1186/s12871-016-0262-6

**Published:** 2016-10-24

**Authors:** C. Steiner, M. P. Steurer, D. Mueller, M. Zueger, A. Dullenkopf

**Affiliations:** 1 Department of Anesthesia and Intensive Care, Kantonsspital Frauenfeld, Postfach Pfaffenholzstr. 4, 8501 Zurich, Switzerland; 2 Department of Anesthesia and Perioperative Care, University of California, San Francisco, USA; 3 Institute of Clinical Chemistry, University Hospital of Zurich, Zurich, Switzerland; 4 Department of Laboratory Medicine, Spital Thurgau, Frauenfeld, Switzerland

**Keywords:** Anesthesia, Midazolam, Anxiolysis, Premedication

## Abstract

**Background:**

Midazolam is commonly used as a pre-anesthesia anxiolytic. It`s elimination may not be fast enough for short procedures. In orally premedicated patients we obtained midazolam plasma concentrations at the end of surgical procedures and compared those to concentrations at anesthesia induction.

**Methods:**

The study was conducted prospectively with consent of the local ethics committee (Ethikkomission Kanton Thurgau, Switzerland) and carried out with written informed consent of each patient. Female patients aged 20 to 60 years undergoing elective procedures with general anesthesia were included, and were divided in two groups according to the planned surgical time: group S (<30 min) and group L (90–120 min), respectively. All patients received 7.5 mg Midazolam po as premedication. Blood samples were drawn at anesthesia induction, and at the end of surgery. Data were compared with t-test (independent samples; significance level *p* <0.05).

**Results:**

Twenty-five patients per group were included. Four patients were excluded from analysis, since midazolam was not detectable in any samples. Time of premedication to the 1st blood sample was not statistically different between groups, neither were Midazolam plasma levels at this time point (*p* = 0.94). None of the patients from group L (*n* = 24), but five patients in group S (*n* = 22) did have a higher plasma level of Midazolam at the end of the case compared to the beginning.

**Conclusions:**

The elimination half-life of oral Midazolam can lead to higher plasma levels at the end of a short procedure compared to those at induction of anesthesia.

**Trial registration:**

German Clinical Trials Register (Deutsches Register Klinischer Studien), DRKS00005429; date of registration 3^rd^ January 2014

## Background

In our institution, for many years Midazolam has been the premedication drug of choice for anxiolysis in patients undergoing general anesthetics.

Generally, the standard dosage for Midazolam used as an oral premedication before general anesthesia is 7.5–15 mg in adults [1]. The drug is usually administered about 30 to 45 min before transporting the patient to the operating room. In clinical practice, this setup has worked in a reliable fashion. Surprisingly, there is no published data on resulting plasma levels of midazolam at induction of anesthesia or at the end of surgical procedures.

This may be an issue because of the rather long elimination half-life of 90–150 min that possibly could result in a comparatively long sedative effect of midazolam. Further, in daily routine, it is not clear, when the peak of absorption actually occurs in relation to the anesthetic process. This can potentially contribute to relatively high plasma levels of midazolam at the end of short procedures and therefore lead to delayed and prolonged emergence from the anesthetic.

In the present study we determined midazolam plasma levels in an everyday clinical setting at the time of induction of anesthesia and at the end of the surgical procedure in procedures of different durations. The study attempted to validate the hypothesis that some patients receiving oral midazolam premedication will show higher plasma levels at the end of short procedures (<30 min) compared to the levels determined just before induction of anesthesia. In contrast, patients undergoing longer procedures (> 1.5 h) will always have lower midazolam levels at the end of anesthesia compared to levels measured just before induction of anesthesia.

## Methods

This study was conducted prospectively with the approval of the local ethics committee (Ethikkomission des Kantons Thurgau, Switzerland; May 2013) and after registration with the German Clinical Trials Register (www.drks.de; DRKS00005429). All study participants were informed beforehand and gave their written consent.

Inclusion criteria were scheduled gynecological surgery under general anesthesia, age between 20 and 60 years, 7.5 mg midazolam po clinically indicated according to the standard of our institution and patient consented prior to the procedure. We excluded patients with the following conditions: allergy or hypersensitivity to benzodiazepines, severe respiratory insufficiency, myasthenia gravis, sleep apnea syndrome, compromised renal and or hepatic function, psychiatric conditions, pregnancy, lactation, alcohol abuse, iv drug abuse, medication with antifungals/ antivirals/ protease inhibitors/ macrolides/ rifampin/ calcium channel blockers/ antihistamines/ St. John's wort/ tranquilizers/ sedatives/ hypnotics/ antidepressants/ antiepileptics in the week before surgery, consumption of grapefruit juice on the day of or before surgery, BMI > 40 kg/m^2^, pre-existing conditions with impaired gastrointestinal absorption, additional midazolam given during procedure.

Patients were fasted 6 hours for solid food and two hours for clear liquid prior to the planned induction of anesthesia.

A total of 50 patients (25 each with a planned operating time of ≤ 30 min (group S), and 90–120 min (Group L), respectively) were given 7.5 mg midazolam po (Dormicum^©^; Roche Ltd., 4253 Reinach, Switzerland) before they were transported to the operating room. The order was called to the ward from the operating room by anesthesia providers that were not part of the study group. In accordance to the standard practice for patients receiving general anesthesia at our institution, the premedication with midazolam po ideally would take place 30 to 45 min before the patient came to the operating room. For the study patients, the time when the po midazolam was given on the ward was recorded.

The anesthesiologist that managed the operating room coordinated the patient flow to the operating room. As a governing principle, we try to have the patient waiting not too long in the operating room holding area; on the other hand we aim to have short room turnover times doing regular overlapping anesthesia inductions. The arrival of the patient to the operating room is routinely documented on the anesthesia record.

On arrival to the operating room, the patient’s level of sedation was assessed by one of the investigators using the OAA/S score (Observer’s Assessment of Alertness/Sedation score; reported as the composite score with OAA/S ranging from 1 [deep sleep] to 5 [alert]) [2]. The OAA/S assesses responsiveness of a given subject to its name, spoken in normal tone, calling loudly or repeatedly, followed by shaking of the subject, respectively. Responsiveness is classified taking into account speech, facial expression and eye opening.

For the general anesthetic, we placed the standard monitoring first (ECG, non-invasive blood pressure measurement, pulse oximetry, BIS [Bispectral index; BIS View, Covidien, Dublin, Ireland; EEG assessment using the bispectral index]), an initial set of values was recorded, before moving on and establishing peripheral venous access on the back of the hand or forearm. While placing the venous cannula, 5 ml of blood were collected in a serum tube. The time of blood collection was recorded (time point 1).

The further course of anesthesia mainly was a Propofol based anesthesia, supplemented with fentanyl and remifentanil. Anesthesia conduct was left to the responsible anesthetist, according to the standard of our institution. At the end of the surgical procedure, before emerging from the anesthesia, a second blood sample (5 ml, serum tubes) was drawn from the opposite arm, and the time was noted (time point 2).

Both serum tubes were labeled and properly stored in the laboratory of our hospital (Kantonsspital Frauenfeld). After completion of the entire study all the samples were sent to the Institute of Clinical Chemistry of the University Hospital Zurich (http://www.usz.ch) where the plasma levels of midazolam were determined by liquid chromatography-mass spectrometry (LC-MS). After addition of stable-isotope labelled internal standards, samples were centrifuged. Twenty microliters of the clear supernatant was submitted to the analysis using a turbulent flow online extraction system. As extraction column, a Cyclone column (Thermo Fisher, Reinach, Switzerland; 50 × 0.5 mm) was used, as analytical column, a Uptisphere C18 (125 × 2 mm). The mobile phases consisted of 10 mM ammonium acetate in water + 0.1 % formic acid and 10 mM ammonium acetate in methanol/acetonitrile 50/50 (v/v) + 0.1 % formic acid. Calibration was done using an in-house prepared six point calibration curve. The method is validated and has an imprecision of less than 5.3 %. The quantification limit is at 0.01 mcmol/l.

On the day after surgery, the patients were asked to rate their level of satisfaction with the overall effect of the preoperative po midazolam on a visual analogue scale (VAS 0-10; 0 = totally dissatisfied, 10 = completely satisfied).

## Statistics

All data (midazolam plasma levels etc.) are presented descriptively as mean ± standard deviation, BIS values and sedation scores are presented as median (minimum - maximum).

Comparisons between group S and group L were made using the t test for independent samples, and using the Mann-Whitney U-test for BIS values and sedation scores.

The statistical evaluations were performed using Microsoft Excel 2010 (Microsoft, Redmond, USA), and StatView 5.0.1 (SAS Institute, Cary NC, USA).

As there are no data about midazolam plasma levels at the end of anesthetics and the main outcome of our study was the number of patients with higher midazolam plasma levels at the end of anesthesia compared to anesthesia induction, there was no formal power analysis performed.

## Results

Fifty patients were enrolled in this study (25 for each group). Four patients (three in group S and one in group L) were excluded from the statistical analysis, because neither midazolam, nor its metabolite 1-OH-midazolam were detectable in any blood samples, and we assumed that the patients have not taken the medication.

The demographic data of the patients are summarized in Table 1. All patients except for one (group L) were classified as ASA physical status I or II.

**Table 1:** Demographic data (mean ± standard deviation); *p* = t-test or Mann-Whitney U-test, as appropriate

	Gruppe S	Gruppe L	*p*
Age (years)	38.5 (± 11.3)	44.4 (± 12.6)	0.08
Height (centimeters)	164.2 (± 4.9)	166.5 (± 6.2)	0.16
Weight (kg)	63.4 (± 11.3)	70.2 (±12.8)	0.21
BMI (kg/m^2^)	23.5 (± 4.4)	25.3 (± 4.5)	0.3
Midazolam dose (mg per kg)	0.12 (± 0.02)	0.11 (±0.02)	0.14
BIS values at arrival in OR (0–100)	94.5 (± 4.1)	95.9 (± 3.2)	0.28
Mean duration of surgical procedure (min)	20.4 (± 12.8)	79.8 (± 36.5)	<0.0001
Level of satisfaction (VAS; 0–10)	8.2 (± 1.8)	8.3 (± 1.6)	0.69

*BMI* Body mass index, *BIS* Bisoectral index, *OR* operating room

The timespan between taking the midazolam po and the 1st blood sample was 63.9 ± 31.4 min in group S and 52.6 ± 18.1 min in group L (*p* = 0.12). In accordance with the study protocol, the time between premedication and the second blood sample was significantly shorter in group S (112.2 ± 35.1 min) than in Group L (165.4 ± 44.5 min; *p* = 0.0002).

The O/AAS values upon arrival in the operating room differed very little between group S (4.2 ± 0.7) and Group L (4.1 ± 0.7; *p* = 0.81) as did the first measured BIS values (94.5 ± 4.1 in group S and 95.9 ± 3.2 in group L; *p* = 0.28).

In the first blood sample the midazolam plasma levels were similar in both groups (0.10 ± 0.07 mcmol/l in group S, 0.10 ± 0.06 mcmol/l in group L; *p* = 0.94). In the second blood sample at the end of the surgical procedure, the midazolam plasma levels in group S (0.06 ± 0.04 mcmol/l) were significantly higher than in group L (0.02 ± 0.02 mcmol/l; *p* = 0.01).

Surgery was exclusively gynecological, comprising mostly curettage, hysteroscopy, and biopsies in Groups S, and vaginal hysterectomy with or without uro-gynecological surgery, and breast surgery in Group L.

**Fig. 1:**
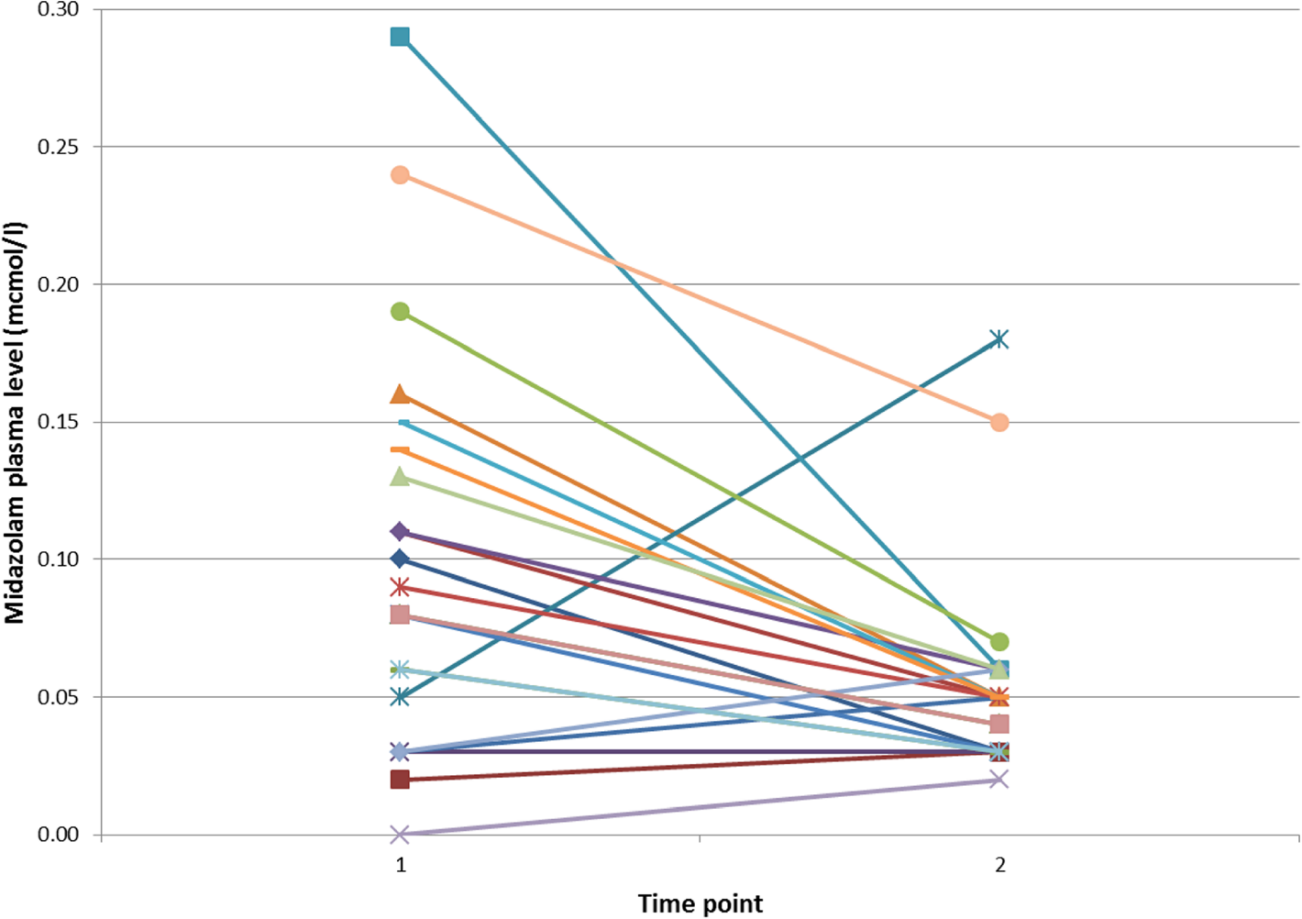
Trend of midazolam plasma levels (mcmol/l) in group S. 1 = before anesthesia induction, 2 = end of surgery

**Fig. 2:**
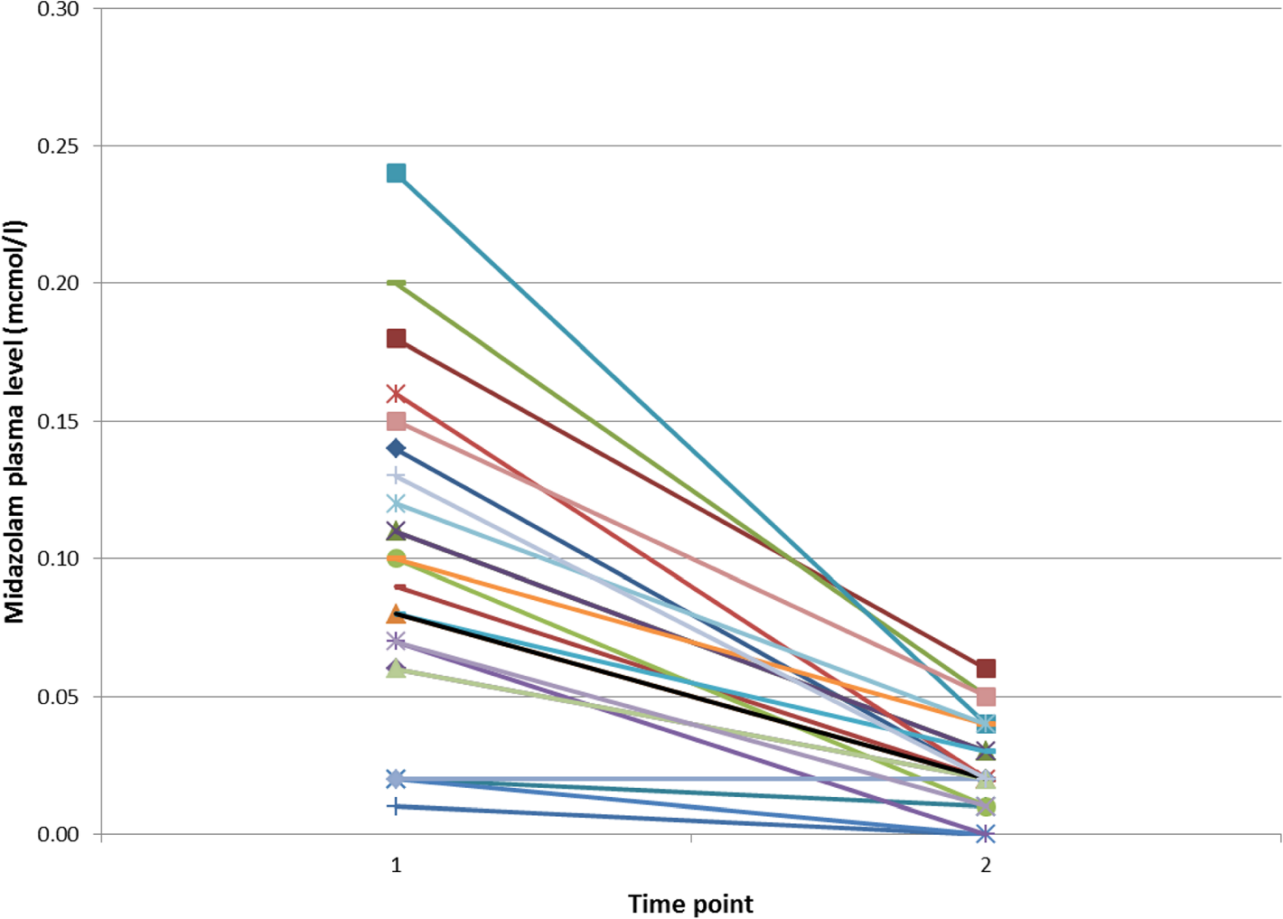
Course of midazolam plasma levels (mcmol/l) in group L. 1 = before anesthesia induction, 2 = end of surgery

In none of the patients in group L (*n* = 24) was the midazolam plasma level higher at the end of surgery than before induction of anesthesia, whereas five patients of the group S (*n* = 22) did have an increased plasma level at the end of the case compared to the pre procedure value (see Figs. 1 or 2).

Excluding the five patients with higher midazolam plasma levels at the end of surgery from group S, plasma levels in group S remained significantly higher than in group L (0.05 ± 0.03 mcmol/l versus 0.02 ± 0.02 mcmol/l; *p* = 0.01).

The satisfaction VAS score did not differ significantly between the two groups (8.2 ± 1.8 in group S and 8.3 ± 1.6 in group L; *p* = 0.69).

## Discussion

In this study, midazolam po pharmaceutical premedication was used in patients undergoing general anesthesia for gynecological procedures. The resulting plasma levels at the time of anesthesia induction were compared to those at the end of the surgical procedure.

The main finding of this study was that in the group of patients that underwent shorter procedures (planned surgical time less than 30 min), a substantial percentage had higher midazolam plasma levels at the end of the operation compared to the time of anesthesia induction. In the group of patients with a longer duration of surgery (90–120 min), all patients had lower midazolam levels at the end of the procedure.

Midazolam serves as a very common choice to achieve anxiolysis in children, adolescents and adults before anesthesia [1, 3, 4]. While children often receive relatively high doses, the standard dosage for adults, if administered po, ranges just around 7.5–15 mg [1]. The reason for this, instead of dosing in relation to body weight might be, that midazolam most commonly is available in tablets of these quantities. However, neither the reliability of midazolam induced sedation, nor the duration of action of midazolam premedication are without debate.

The optimal time point for the administration and dosing of midazolam preoperatively remains unclear and appears to be individually different. According to the study by Lim the sedative effects started to wear off after 45–60 min from the time point when midazolam was given as a tablet [5].

In our study it took 48 min from the time of application of the drug to the first pre-operative blood sample. For some individual patients the peak sedative effect could have already happened before they actually came to the operating room.

In addition, the interindividual metabolism of midazolam can differ greatly. After oral administration of 15 mg po in young, healthy people its bioavailability varies between 30 and 50 %, and the elimination half-life is between 1.5 and 2.5 h [6]. A comparative study with participants from five different ethnic Chinese groups also demonstrated large differences between these [7]. So, apart from the fixed dose, differences in timing of anesthesia premedication, varying resorption and metabolsing of the drug, as well as genetic factors contribute to the high variability of resulting plasma concentracions seen in this study under clinical routine conditions.

The patients in our study were consistently medicated with 7.5 mg midazolam po. As this study was designed to assess clinical routine, this fixed dose was chosen, instead of dosing in relation to body weigth. This resulted in moderate midazolam plasma levels. The reported mean of 0.11 (± 0.06) micromol/l is corresponding to about 30 ng/ml. In the Swiss Drugs registry (http://www.compendium.ch), 7.5 to 15 mg midazolam PO is given as the standard dosing for anesthesia premedication. The laboratory of our institution regards 0.3 to 1 micromol/l as therapeutic plasma level, however without differentiating for which indication. Accordingly, both the subjectively and objectively detectable sedative effect preoperatively was only moderate, but at the same time the subjective satisfaction was rather good. OAA/S scores and BIS values at arrival in the operating room showed only a narrow variation, meaning the effect of the premedication being fairly predictable.

Even when higher doses are given, the sedative effect of midazolam often appears to be only modest and/or hard to quantify both in older children and adults on arrival to the operating room and immediately before anesthesia induction. Brosius et al. describe adolescents who received 20 mg midazolam po, as having a BIS value of 92 immediately before induction of anesthesia with an OAA/S sedation score indicating a relevant level of sedation in only 40 % of the patients [4]. This may partially be explainable by the fact that entering the operating room and attaching the monitoring to the patient with the imminent beginning of anesthesia and surgery probably represents the moment of greatest stress and nervousness. Oral intake of midazolam as a tablet also leads to a lower sedative effect compared to sublingual application of the same dose, because of bypassing the first-pass effect in the liver. Various drug compounds are also likely to play a role. Lim et al. have shown that it can take up to 20 min, until a Dormicum^©^ tablet is completely dissolved in the mouth [5].

The plasma levels that we measured seem very low compared to what is reported in pediatric anesthesia publications. However, for example in Brosius and Bannister’s study, resulting in much higher plasma levels, adolescent patients were only about 10 % lighter on average and received almost a 3-fold dose of midazolam than compared to our setting [4].

All the above mentioned points add to our main finding that under clinical routine conditions some patients having been orally premedicated with Dormicum^©^ before short surgical procedures, had higher plasma levels of the drug at the end of their procedure (when expected to awake from the anesthetic) compared to the induction of anesthesia when anxiolysis and sedation were most desirable.

Brosius and Bannister were not able to show an interaction of midazolam with general anesthetics (Sevoflurane-based) in their setting and the recovery period after a standardized anesthetic in their midazolam group compared with a placebo group was not significantly longer [4, 8]. However, in settings like e.g. ambulatory or office based anesthetics, the rather long half-life of midazolam induced sedation might be of concern. If only intended for providing preoperative anxiolysis and not added as medication for maintaining the anesthetic, a reliably performing, shorter acting anxiolytic could be a more suiting companion for this purpose. If preferring a benzodiazepine, remimazolam may in the future be an interesting option [9].

Our study setup had some limitations. The actual intake of the midazolam tablet on the ward happened largely unsupervised. The fact that four patients had no detectable plasma levels for neither midazolam nor its metabolites could be the result from the patients not having actually taken the tablet. It is also conceivable that some patients may have ingested only fractions of the actual tablet. Furthermore, it would have been very interesting to correlate the midazolam plasma levels at the end of the procedure with the time it took to emerge from anesthesia. In order to do so, the anesthetic management would have needed standardization with a protocol, which was not the case in our study. Also, if correlating midazolam plasma levels to clinical effect, it might be necessary to consider levels of α-hydroxy-midazolam, a metabolite with intrinsic action. However, the midazolam plasma levels in our study were quite low, and therefore most probably not significantly influencing emerging times after anesthesia.

Finally, dosing in relation to body weight might lead to more reliable plasma concentrations, but is not practicable with standard Dormicum^©^ tablets.

The results of this study might lead the anesthesiologist to reconsider the use of standard, rather long acting midazolam premedication in patients undergoing surgery with a planned duration of less than 30 min. Non-pharmacological anxiolysis or new, shorter acting substances may be the solution to this problem.

## Conclusions

In summary, the most pertinent finding in this study was the fact that midazolam po as an anxiolytic before general anesthesia in short interventions can result in higher plasma levels at the end of the procedure.

## Abbreviations

BIS: Bispectral index
BMI: Body mass index
OAA/S score: Observer’s Assessment of Alertness/Sedation score
VAS: Visual analogue scale
